# Protein kinase CK2 modulates HSJ1 function through phosphorylation of the UIM2 domain

**DOI:** 10.1093/hmg/ddw420

**Published:** 2016-12-28

**Authors:** Daniele Ottaviani, Oriano Marin, Giorgio Arrigoni, Cinzia Franchin, Jordi Vilardell, Michele Sandre, Wenwen Li, David A. Parfitt, Lorenzo A. Pinna, Michael E. Cheetham, Maria Ruzzene

**Affiliations:** 1 Department of Biomedical Sciences, University of Padova, Via U. Bassi 58/b 35131 Padova, Italy; 2 Proteomics Center, University of Padova and Azienda Ospedaliera di Padova, Via G. Orus 2/B, 35129 Padova, Italy; 3 UCL Institute of Ophthalmology, 11-43 Bath Street, London EC1V 9EL, UK

## Abstract

HSJ1 (DNAJB2), a member of the DNAJ family of molecular chaperones, is a key player in neuronal proteostasis maintenance. It binds ubiquitylated proteins through its Ubiquitin Interacting Motifs (UIMs) and facilitates their delivery to the proteasome for degradation. Mutations in the *DNAJB2* gene lead to inherited neuropathies such as Charcot-Marie-Tooth type-2, distal hereditary motor neuropathies, spinal muscular atrophy with parkinsonism and the later stages can resemble amyotrophic lateral sclerosis. HSJ1 overexpression can reduce aggregation of neurodegeneration-associated proteins *in vitro* and *in vivo*; however, the regulation of HSJ1 function is little understood. Here we show that CK2, a ubiquitous and constitutively active protein kinase, phosphorylates HSJ1 within its second UIM, at the dominant site Ser250 and the hierarchical site Ser247. A phospho-HSJ1 specific antibody confirmed phosphorylation of endogenous HSJ1a and HSJ1b. A tandem approach of phospho-site mutation and treatment with CK2 specific inhibitors demonstrated that phosphorylation at these sites is accompanied by a reduced ability of HSJ1 to bind ubiquitylated clients and to exert its chaperone activity. Our results disclose a novel interplay between ubiquitin- and phosphorylation-dependent signalling, and represent the first report of a regulatory mechanism for UIM-dependent function. They also suggest that CK2 inhibitors could release the full neuroprotective potential of HSJ1, and deserve future interest as therapeutic strategies for neurodegenerative disease.

## Introduction

HSJ1 (also known as DNAJB2) is a key component in neuronal protein quality control. It is a molecular chaperone that is preferentially expressed in neurons that can act as a shuttling factor to sort chaperone clients to the proteasome (1). *DNAJB2* mutations have been identified as causative of hereditary neuropathies. The first finding was in a family with a rare autosomal recessive distal hereditary motor neuropathy (dHMN) where a splice site mutation in the *DNAJB2* gene was found (2). The phenotypic spectrum of *DNAJB2-*related neuropathies was later broadened by the discovery of two additional mutations of the *DNAJB2* gene in patients with dHMN and Charcot-Marie-Tooth disease type 2 (CMT2) (3). A further case of dHMN, parkinsonism and cerebellar ataxia due to HSJ1 mutation was recently reported (4), and a large *DNAJB2* gene deletion was found in a family with recessive spinal muscular atrophy and parkinsonism (5).

**Figure 1.**
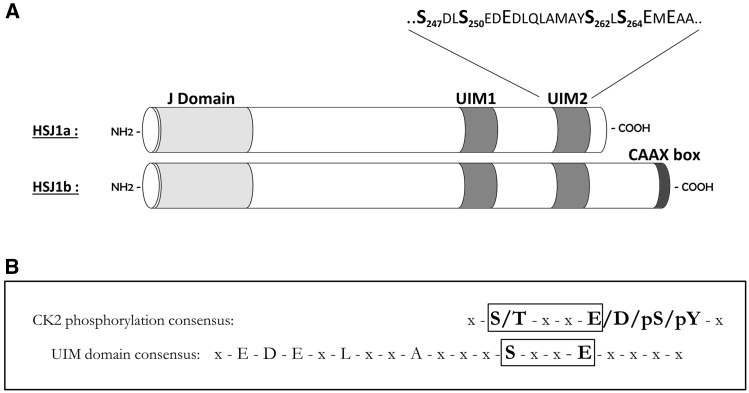
CK2/UIM consensus and HSJ1 structure organization. (**A**) The consensuses for phosphorylation by CK2 (19,20) or for Ub-protein binding by UIM (18) are shown. The overlapping segments are boxed. (**B**) The domains organization of the two HSJ1 isoforms are shown as in (1,54). The sequence of UIM2 is also reported, highlighting the CK2 putative sites.

Two HSJ1 isoforms are expressed in humans, HSJ1a and HSJ1b, as a result of alternative splicing (6). They share the same basic domain structure (Fig. 1A), but their intracellular localization differs: HSJ1a is cytosolic and nuclear, while HSJ1b has a longer C-terminus and is anchored to the cytoplasmic face of ER due to C-terminal geranylgeranylation (7). At the N-terminus, HSJ1 presents the typical J domain of DnaJ (Hsp40) molecular chaperone family members (8), that stimulates substrate loading onto Hsp70 chaperones (1,9). Near the C-terminus HSJ1 has two Ubiquitin Interacting Motifs (UIMs), that function to bind ubiquitin chains and help prevent client protein aggregation. The neuroprotective role of HSJ1 has been demonstrated in different disease models (10): it suppresses the aggregation of polyglutamine expanded proteins, significantly enhancing mutant huntingtin solubility in Huntington disease in cells and in mice (1,11), and promoting misfolded protein targeting to the ubiquitin-proteasome system (12); HSJ1a cooperates with Hsp70 to promote proteasome-degradation of ataxin-3, a protein responsible for spinocerebellar ataxia type 3 (SCA3) (13); HSJ1a prevented the aggregation of the misfolded C289G Parkin, a Parkinson disease-associated ubiquitin-protein ligase mutant, and restored its function in mitophagy (14). Interestingly, a protective function of HSJ1a has also been demonstrated in ALS models: its overexpression in motor neurons of SOD1^G93A^ mutant transgenic mice was found to improve the disease symptoms; the molecular mechanism was related to HSJ1a association with SOD1, with its consequent increased ubiquitylation and reduced aggregation (15). More recently HSJ1a was shown to be highly effective at preventing the aggregation of TDP-43 (16). Therefore, HSJ1 functions to regulate the proteasomal targeting of misfolded proteins, and protect neurons against cytotoxic protein aggregation by the coordinate actions of its J and UIM domains.

UIMs are sequences of about 20 amino acids, present in proteins belonging to the proteasomal and lysosomal protein degradation systems, where they confer the ability to bind mono- and/or poly-ubiquitylated proteins (17). A conserved sequence has been identified on UIMs as the minimal consensus for the Ub-binding function (18). This sequence includes a Serine residue followed by an acidic residue three positions downstream. We noticed that this motif is superimposable to the consensus sequence for the recognition and phosphorylation by protein kinase CK2 (Fig. 1B). CK2 is an acidophilic kinase that prefers Ser/Thr sites surrounded by numerous acidic residues. Its consensus sequence is one of the most specific and well-defined amongst protein kinases (19,20): the minimal requirement is exactly represented by an acidic determinant at position +3 downstream to the target site. CK2 is a highly pleiotropic and constitutively active enzyme mainly present in cells as a tetramer, composed of two catalytic (α and/or α’) and two regulatory (β) subunits. It phosphorylates hundreds of substrates (21) and controls many cellular processes, but its major recognized function is in counteracting apoptosis (22–24). Although present in all cells, CK2 is overexpressed in cancers, and is currently considered a valuable target in anticancer therapies (25). However, the multi-faceted profile of CK2 points for its involvement in a broader range of human diseases, such as neurodegeneration, where its importance has been already suggested, although never studied in detail.

Here we investigate the connection between CK2 and HSJ1 as a paradigmatic example of functional cross-talk between the phosphorylation and ubiquitin dependent signalling, based on the working hypothesis that phosphorylation of the serines conforming to the CK2 consensus on the HSJ1 UIM2 may affect its ability to bind ubiquitylated proteins.

## Results

### HSJ1 phosphorylation by CK2

**Figure 2.**
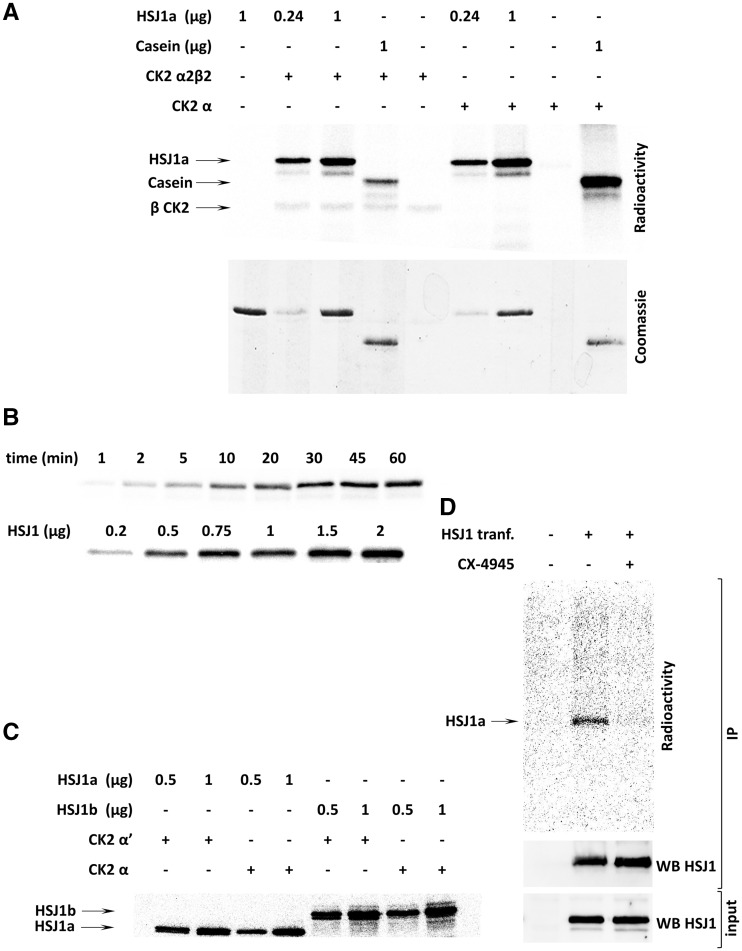
HSJ1 phosphorylation by CK2 *in vitro* and in cells. (**A**) Different amounts of recombinant HSJ1a were incubated with a radioactive phosphorylation mixture in the presence of CK2 α2β2 (50 ng) or α (50 ng), as indicated. Casein was used as a control substrate for CK2 activity. (**B**) Phosphorylation of HSJ1 by α CK2 (50 ng) was performed for increasing times (upper panel, 0.5 µg HSJ1) or with increasing HSJ1 amount (lower panel, 5 min incubation). The radioactivity is shown. (**C**) Different amounts of recombinant HSJ1a or HSJ1b were phosphorylated by CK2 α (50 ng) or α’ (50 ng), as indicated. The radioactivity is shown. (**D**) HEK-293T cells were transfected with myc-HSJ1a (+) or empty vector (−), then loaded with (^32^P)phosphate, and treated with 10 µM CX-4945, as indicated. Anti-myc IP was performed, followed by analysis of radioactivity and of amount of HSJ1a immunoprecipitated. The lower panel (input) shows 20 µg of total protein lysate, analysed by WB for HSJ1 before IP. Radioactive phospho-proteins were separated by SDS-PAGE or SDS-PAGE/blot and analysed for radioactivity using Cyclone Plus (PerkinElmer), or by Coomassie blue staining, or by WB, as indicated in each panel. Representative experiments are shown.

To test the hypothesis that HSJ1 is phosphorylated by CK2, we first analysed *in vitro* phosphorylation with recombinant human HSJ1a as substrate in radioactive phosphorylation assays. In the presence of monomeric CK2 (α catalytic subunit) or CK2 holoenzyme (α_2_β_2_), we observed a remarkable phosphorylation (Fig. 2A) that was concentration and time dependent (Fig. 2B). As expected, the other HSJ1 isoform, HSJ1b, which shares the same CK2 putative sites with HSJ1a, was also readily phosphorylated by CK2 *in vitro* (Fig. 2C). The two isoforms of the CK2 catalytic subunits, α and α’, had a similar activity towards HSJ1 (Fig. 2C).

To confirm HSJ1 phosphorylation in the cellular milieu, we transiently expressed myc-tagged HSJ1a in HEK-293T cells, and performed HSJ1 immunoprecipitation after cell loading with [^32^P]phosphate to generate an intracellular pool of radioactive ATP. The autoradiography analysis of the immunoprecipitates confirmed that HSJ1 undergoes phosphorylation in cells (Fig. 2D); moreover, we observed a strong reduction of phosphate incorporation upon cell treatment with the CK2 specific inhibitor CX-4945 (26), suggesting that CK2 is a prominent HSJ1 phosphorylating kinase.

### Identification of HSJ1 phospho-site(s)

In order to identify the precise phosphorylated residue(s) in HSJ1, HSJ1a was phosphorylated *in vitro* by CK2, digested with V8 protease and the proteolytic peptides analysed by mass spectrometry (MS/MS). This identified Ser247, Ser250, and Ser264 as phospho-amino acids (Supplementary Materials, Table S1 and Fig. S1).

**Figure 3.**
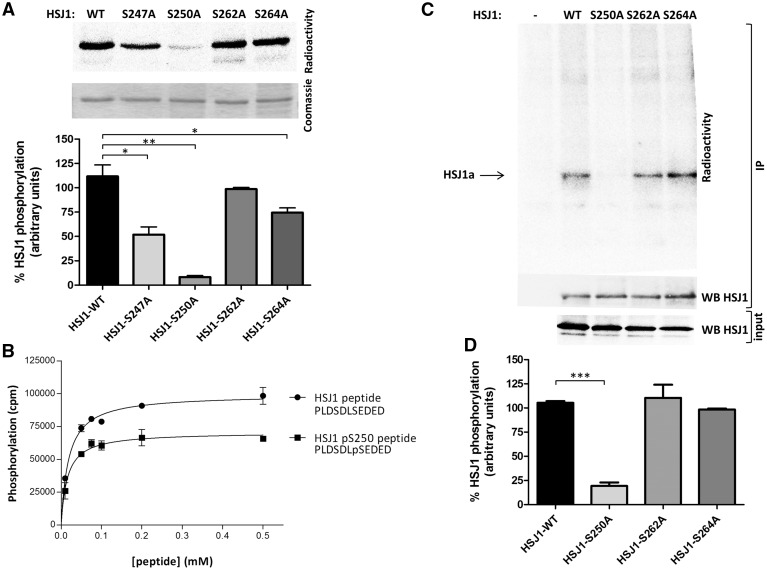
Phosphorylation of HSJ1 WT and mutants by CK2. (**A**) Recombinant WT or mutant HSJ1 proteins were phosphorylated by CK2 α2β2 (25 ng) and separated by SDS-PAGE. Radioactivity and Coomassie staining are shown of representative experiments. The bar graph shows quantification of radioactive (mean of *n*=3 experiments ± SEM. *P<0.05, **P<0.001, unpaired, two-tailed, Student's *t*-test). (**B**) Increasing concentrations of the indicated peptides were phosphorylated by CK2 α2β2 (25 ng) and analysed for the incorporated radioactivity. More details under Materials and Methods. (**C**) anti-myc IP was performed from lysates of HEK-293T cells transfected with myc-HSJ1a WT or mutants, or empty vector (−), pre-loaded with [^32^P]phosphate. The radioactivity and the amount of HSJ1a in immunoprecipitates and in total lysates (20 µg) are shown (representative experiments). (**D**) Bar graph shows quantification of radioactive bands of experiments as in panel C, normalized to the amount of immunoprecipitated HSJ1a (mean of *n*=4 experiments ± SEM. ***P≤0.001, unpaired, two-tailed, Student's *t*-test).

To validate these findings, we produced Ser-to-Ala single-site mutants of all the identified sites, and also the putative additional CK2 site Ser262 within the second UIM, although it was not identified by MS/MS. *In vitro* phosphorylation of the recombinant purified HSJ1a mutants was consistent with the MS outcomes (Fig. 3A), supporting the view that Ser262 is not affected; Ser264 very weakly contributes to the overall phosphorylation, while Ser250 appears to be the main phosphorylation site, as its substitution almost completely abrogated phosphorylation. A little surprisingly, Ser247 mutation to Ala caused an about 50% drop on the total HSJ1a radioactivity. A plausible explanation is that although Ser247 does not fulfill the CK2 minimal consensus sequence, because it does not have an acidic residue in +3 position, once Ser250 is phosphorylated the residue becomes acidic and fits the consensus sequence. This implies that a hierarchical phosphorylation can occur in HSJ1, where the previous phosphorylation of Ser250 primes Ser247 for subsequent phosphorylation. This hypothesis was supported by synthetic peptides reproducing the HSJ1 sequence 244–254 in two variants, differing by the presence or absence of phosphate at Ser250. As shown in Fig. 3B, both peptides are readily phosphorylated by CK2, with a superiority of the one presenting both Ser247 and Ser250 as available sites.

The results obtained *in vitro* with the recombinant mutants were confirmed in cells: expression of the mutants in HEK-293T followed by their immunoprecipitation from [^32^P]phosphate pre-loaded cells, revealed that the Ser250Ala mutant was not phosphorylated, demonstrating that this site is the critical residue for the phosphorylation of the HSJ1 protein (Fig. 3C).

### HSJ1 phospho-specific antibodies

**Figure 4.**
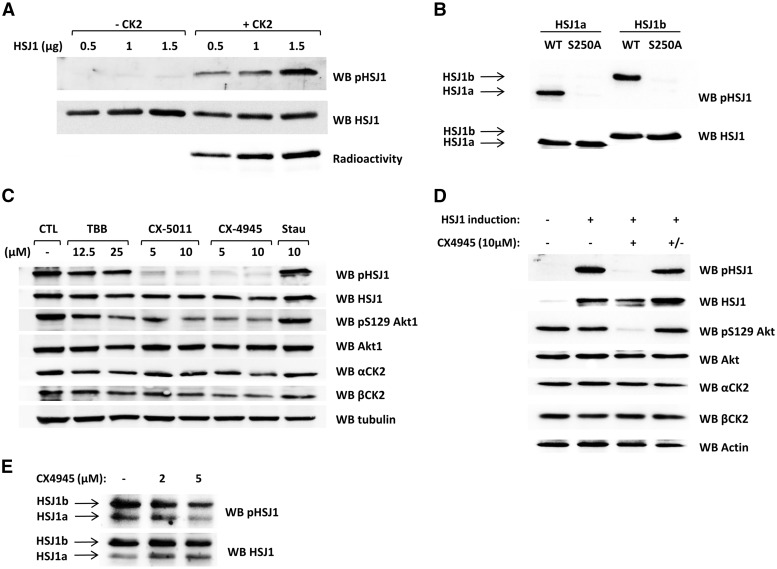
CK2-dependent HSJ1 phosphorylation detected *in vitro* and in cells by means of phospho-specific antibodies. (**A**) Increasing amount of recombinant HSJ1a were incubated in the absence or in the presence of CK2 α2β2 (25 ng) and a radioactive phosphorylation mixture; samples were analysed by WB with the phospho-specific antibody or with total HSJ1 antibody. The radioactivity of the bands is also shown. (**B**) WT or mutant HSJ1 isoforms were expressed in HEK-293T cells. 20 µg of total lysate proteins were analysed by WB with the phospho-specific antibody or with total HSJ1 antibody. (**C**) HEK-293T cells transfected with WT HSJ1a were treated for 3 h with the indicated concentrations of kinase inhibitors; 20 µg of total lysate proteins were analysed by WB, as indicated. (**D**) GFP-HSJ1a expression was induced in CHO cells by 3 µg/ml tetracycline. Where indicated (+) cells were treated for 3 h with CX-4945; +/− refers to cells that where incubated with CX-4945 for 3 h, then the inhibitor was removed and cells incubated for further 3 h before lysis. 20 µg of total lysate proteins were analysed by WB as indicated. HSJ1 expression was detected by means of anti-GFP WB. (**E**) 25 µg of total protein lysates from human BJ fibroblasts were resolved by SDS-PAGE followed by immunoblotting to assess endogenous HSJ1 phosphorylation. Where indicated, cells were treated for 2 h with CX-4945. WB analyses were performed, as indicated.

Collectively, these data indicate that there are two major phospho-sites in the HSJ1 protein, at Ser250 and Ser247, with a minimal contribution of other sites. We therefore decided to produce phospho-specific antibodies towards these sites, choosing a strategy that allows the detection of the protein either when mono-phosphorylated on Ser250 or bi-phosph�orylated at Ser250 and Ser247. The antibodies were first validated *in vitro* toward the protein phosphorylated by recombinant CK2 (Fig. 4A), and by Surface Plasmon Resonance (SPR) experiments, these data show a high affinity for the mono-phosphorylated pSer250 peptide, while a bis-phosphorylated peptide reproducing pSer247/pSer250 sequence was more poorly recognized (Supplementary Material, Fig. S2A). Then we used the phospho-specific antibodies to assess the HSJ1 phosphorylation in cells. First, we expressed either the wild-type (WT) protein or the Ser250 mutant (S250A) of HSJ1a or b in HEK-293T cells. The WT protein was strongly detected by the phospho-specific antibody, while the mutation of Ser250 completely abrogated the signal (Fig. 4B). A major decrease in the phospho-antibody signal was observed in cells expressing WT HSJ1a and treated with a panel of CK2 inhibitors (Fig. 4C). The strong reduction in HSJ1 phosphorylation in response to CX-4945 or CX-5011 (27) was less evident with TBB, but was consistent with the reduction of immunoreactivity for the well-known CK2 target Akt pSer129 (28), or even more pronounced. Furthermore, staurosporine, which inhibits most protein kinases, but not CK2 (29), had no effect on pHSJ1 imm�unoreactivity.

Then we tested the phospho-HSJ1 antibodies on a CHO cell line with inducible HSJ1 overexpression. In this case we also observed a significant level of phosphorylation, which was sensitive to CK2 inhibition (Fig. 4D). As expected, the removal of the inhibitor rapidly restored the HSJ1 phosphorylation, due to the short half-life of the CX4945-CK2 complex, as recently described (30).

To assess if phosphorylation also occurs on endogenous HSJ1, we used human fibroblasts that have been reported to express detectable levels of endogenous HSJ1 (2,3). We found that endogenous HSJ1 was phosphorylated in a CK2-dependent manner, as judged by anti-phospho-HSJ1 reactivity and sensitivity to CK2 inhibition (Fig 4E).

### Effects of phosphorylation on HSJ1 function

**Figure 5.**
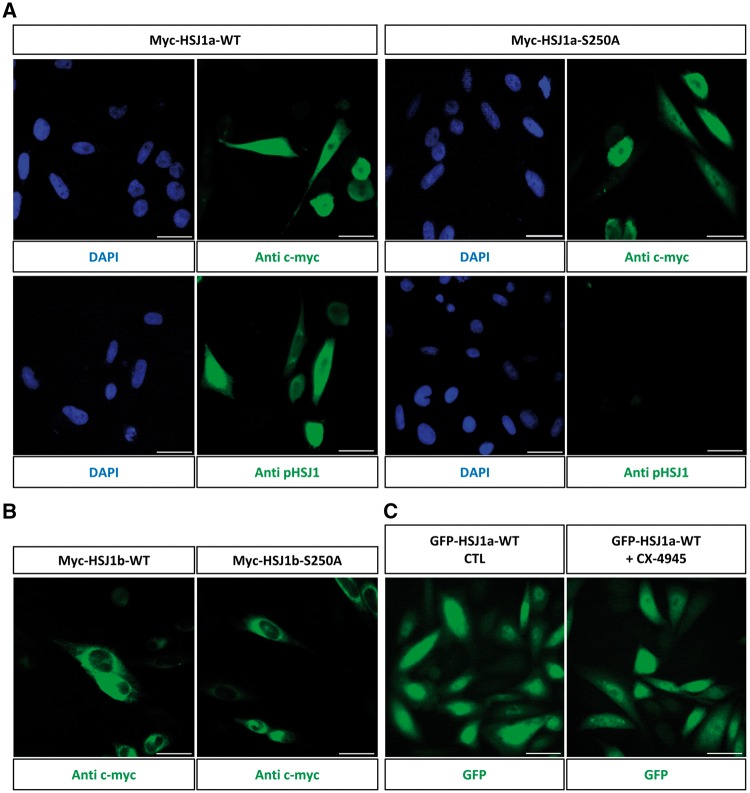
Subcellular localization of HSJ1 and phospho-HSJ1. (**A**) SK-N-SH cells transfected with myc-HSJ1a WT or Ser250Ala mutant were stained with anti-c-myc or anti-phospho HSJ1 antibody, as indicated, and detected by the Alexa Fluor 488 secondary antibody. Nuclei were stained with DAPI. (**B**) SK-N-SH cells transfected with myc-HSJ1b WT or Ser250Ala (S250A) mutant were stained with anti-c-myc antibody and revealed by the Alexa Fluor 488 secondary antibody. (**C**) GFP-HSJ1a expressing CHO cells were treated for 3 h with 10 µM CX-4945 and analysed for fluorescence localization. Scale bars = 50 µm.

To test the hypothesis that phosphorylation of HSJ1 could alter its subcellular localization, as reported for the Ub-binding protein ataxin-3 (31), immunofluorescence analysis was performed with the phospho-HSJ1 antibodies on myc or GFP tagged HSJ1 in the neuronal cell line SK-N-SH; however, we observed a very similar pattern for total HSJ1a or phospho-HSJ1a signal (Fig. 5). Furthermore, transfection of the Ser250Ala instead of WT HSJ1 did not alter the localization observed with a pan-HSJ1 antibody, while abrogating the phospho-specific signal (Fig. 5A). In all cases the HSJ1a signal was predominantly in the cytosol and nucleus, as previously reported (7). Even in the case of HSJ1b, mutation of the phosphorylation site did not alter the localization of the WT protein, which was consistent with its published localization on the cytoplasmic face of the ER (7) (Fig. 5B). The lack of effect of phosphorylation on subcellular HSJ1 localization was also confirmed by treatment of HSJ1-inducible cells with CX-4945, which had no overt effect (Fig. 5C).

These data show that HSJ1 is phosphorylated within its second UIM domain, and that CK2 is the main responsible kinase.

**Figure 6.**
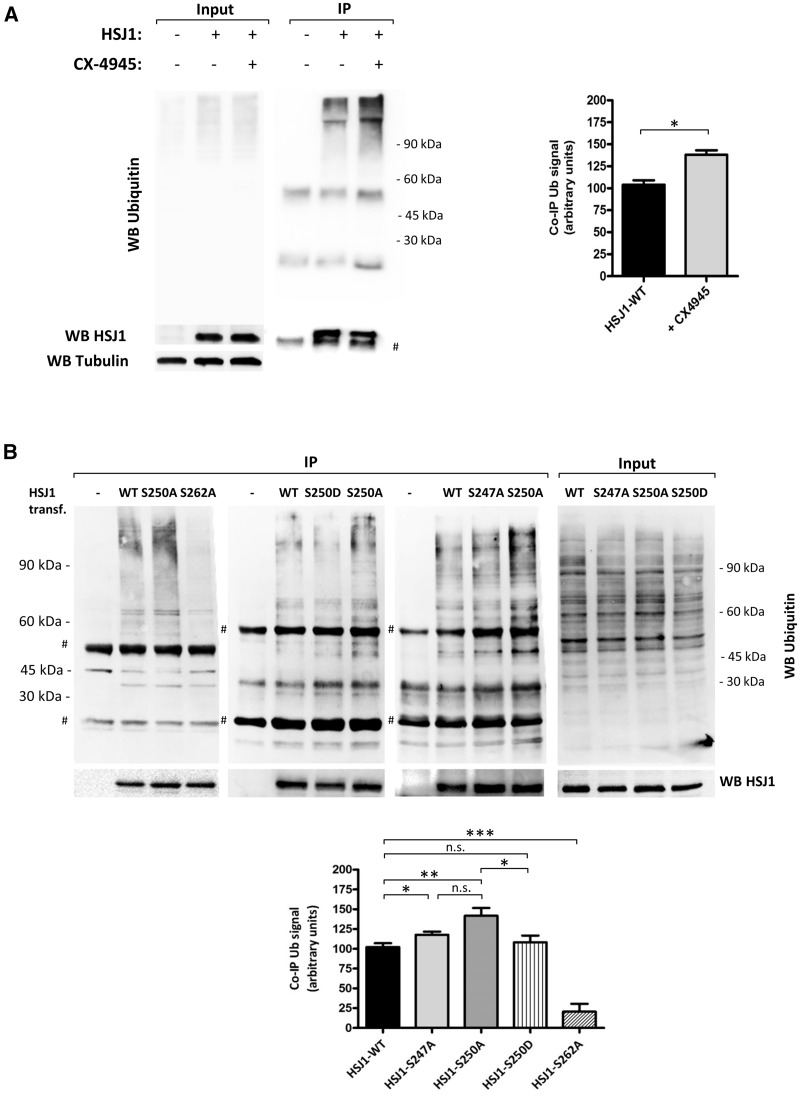
Effects of HSJ1 phosphorylation on Ub-client binding. (**A**) 1 mg of protein lysates from CHO cells stably transfected with HSJ1 (+) or not (-) were used for HSJ1 immunoprecipitation. Where indicated, cells were treated with 10 µM CX-4945 for 3 h before lysis. 20 µg lysate proteins were loaded for input analysis. Mw marker migrations are indicated on the right. # indicates immunoglobulin bands. Bar graph shows the quantification of the Ub signals assigning 100 to the signal of Ub-proteins co-IP with WT HSJ1. Data are presented as mean of *n*=3 experiments ± SEM. *P<0.05, unpaired, two-tailed, Student's *t*-test. (**B**) HEK-293T cells were transiently transfected with empty vector (−) or HSJ1 WT or mutants, as indicated; 600 µg proteins from cell lysate were used for HSJ1 immunoprecipitation. Analysis was performed by WB for Ubiquitin and for HSJ1. # indicates immunoglobulin bands. Mw marker migrations indicated on the left refer to the most left panel, on the right to the three right panels. Bar graphs shows the quantification of the Ub signals assigning 100 to the signal of Ub-proteins co-IP with WT HSJ1. Data are presented as mean of *n*=4 experiments ± SEM. *P<0.05 **P<0.01 ***P<0.001, n.s., not significant, unpaired, two-tailed, Student's *t*-test.

The second UIM domain of HSJ1 has been reported to be the most important in the binding of ubiquitylated proteins (1), therefore we wanted to test the hypothesis that the phosphorylation could alter HSJ1 client protein recognition (1). Co-immunoprecipitation was used to analyse the amount of HSJ1a-associated ubiquitylated-proteins (Ub-proteins) and how this was affected by HSJ1 phosphorylation state. Initially, we assessed the effect of CK2 inhibition on HSJ1a stable-inducible cells (Fig. 6A): importantly, more Ub-proteins were co-precipitated with HSJ1a when cells were treated with CX-4945, which did not change the total amount of Ub-proteins. To test whether this was due to the reduced HSJ1 phosphorylation or to other effects of CK2 inhibition, we applied immunoprecipitation of HSJ1 from HEK-293T cells overexpressing HSJ1a WT or phospho-mutant forms (Fig. 6B). We also exploited the mutant of Ser262, which is not a CK2 site, but is known to impair UIM functionality (1), and therefore was used as negative control. As expected, the Ser262Ala mutant co-precipitated with the lowest amount of Ub-proteins, the phospho-null mutant Ser250Ala showed enhanced binding to Ub-clients compared to WT HSJ1. By contrast, the same site Ser250 mutated to Asp, which mimics phosphorylation, was similar to WT or even less effective for Ub-client association, while the Ser247Ala mutation produced an intermediate level of the coIP Ub signal. These results indicate that the phosphorylation of HSJ1 by CK2 reduced its ability to bind ubiquitylated clients.

**Figure 7.**
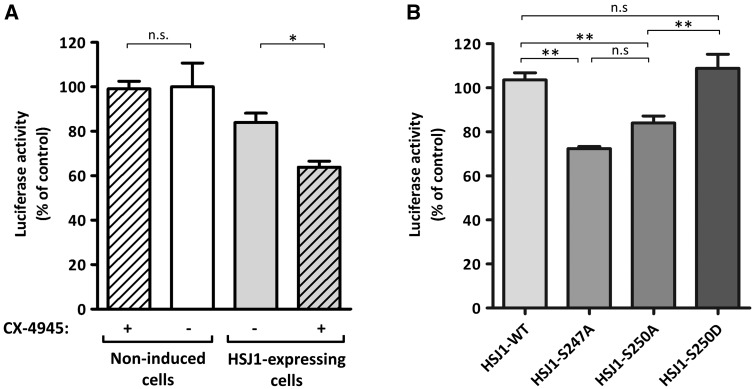
Effects of HSJ1 phosphorylation on its chaperone activity. (**A**) Luciferase was expressed in HSJ1 stable transfected CHO cells; HSJ1 expression was induced as indicated. Where indicated, cells were treated for 3 h with 10 µM CX-4945. (**B**) SK-N-SH cells were co-transfected with HSJ1 WT or mutants and luciferase before activity measurements. Data are presented as mean of *n*=4 (A) or *n*=3 (B) replicates ± SEM. Statistical significance was determined by unpaired, two-tailed, Student's *t*-test. *P<0.05; **P<0.01, n.s., not significant.

Finally, we tested the hypothesis that phosphorylation could affect the HSJ1 chaperone activity. It has been shown that HSJ1 associates with luciferase, and promotes a decrease of the luciferase level/activity in a UIM dependent manner (12). With this in mind, we expressed luciferase in control (uninduced) cells and in HSJ1 induced cells, and we treated them with CX-4945. Luciferase activity was reduced in cells expressing HSJ1a (as expected with the proteasomal targeting of the enzyme), and this was further reduced by the presence of the CK2 inhibitor, which had a negligible effect in cells not expressing HSJ1a (Fig. 7A). We then compared HSJ1a WT and mutants for their effect on co-expressed luciferase. Cells expressing the HSJ1 phospho-site mutants Ser247Ala and Ser250Ala displayed the lowest luciferase activity, while the phospho-mimetic Ser250Asp mutant produced an effect similar to the WT (Fig. 7B). Collectively, these data indicate that the phosphorylation by CK2 prevents the full functionality of the HSJ1 chaperone activity.

## Discussion

Aberrant signal transduction is a major cause of human diseases. In healthy conditions, several types of post-translation modifications are coordinated in a network of interconnections that is essential to correctly tune a signal. In this frame, an emerging theme is represented by the multiple connections between phosphorylation and ubiquitylation/proteostasis (32). Here we disclose a novel crosstalk between these two signalling systems, represented by the kinase CK2 and the Ub-binding protein HSJ1, whose defective function is related to inherited neuropathies.

CK2 has been frequently connected to protein degradation pathways. It has a well-defined role in the regulation of caspases, mainly through the phosphorylation of crucial sites in caspase substrates that become refractory to proteolysis (23). CK2 involvement in the regulation of other proteolytic pathways has been also reported. Interestingly, it has been demonstrated that the SUMO interacting motifs (SIMs), when phosphorylated by CK2, display a higher affinity for the Ub-like molecule SUMO. This was initially reported for the tumour suppressor PML, the exosome component PMSCL1, and the E3 SUMO ligase PIAS1 (33), and recently for the RAP80 protein, a key component of double-strand DNA break repair mediated by the BRCA1 complex (34). CK2 also phosphorylates p62, an autophagy-related Ub-binding protein; the phosphorylation occurs at several sites, also within the UBA (Ub-associated) domain of p62, and promotes a shift of specificity that allows the binding to both K63-linked and K48-linked poly-Ub (35). CK2 has been also reported to regulate the clearance of misfolded proteins by recruiting them to aggresomes in response to stress (36). Moreover, the Ub-interacting protein Ataxin-3 was found phosphorylated by CK2 at several sites; although phosphorylation did not affect protein interactions, it affected its subcellular localization and stabilization (31).

To the best of our knowledge, it has never been reported before that CK2 phosphorylates a protein within a UIM sequence, with functional consequences for Ub-client binding. Here we show that HSJ1, a protein involved in the delivery of Ub-proteins for proteasomal degradation, is phosphorylated by CK2 on its second UIM, both *in vitro* and in cells, and this inhibits its function. Other findings have previously connected CK2 to the proteasome function. Tsuchiya and coworkers showed that CK2, by inhibiting the transcriptional activity of Nrf1, down-regulates the expression of proteasome subunit genes, and that CK2 knockdown alleviates the accumulation of Ub-proteins upon proteasome inhibition (37). Moreover, CK2 controls the stability of 26S proteasome by phosphorylating the 20S proteasome alpha subunit C8 (38). A recent proteomics study from our laboratory showed that cell treatment with the CK2 inhibitor quinalizarin is accompanied by a dramatic increase of all the components of the proteasomal catalytic core (39). Other studies have correlated the activity of CK2 to the proteasomal degradation of specific proteins. It is worth mentioning the direct phosphorylation of PML by CK2 at Ser517, which promotes the proteasome-dependent degradation of PML, thus preventing its tumour-suppressive activity (40). More recently, it was demonstrated that the SUMOylation of PML is required for its interaction with CK2 and subsequent proteasome-mediated degradation (41). PTEN is another tumour suppressor protein degraded by proteasome and phosphorylated by CK2; in this case, the phosphorylation protects the protein from degradation, although, at the same time it inhibits its function (42). Similarly, Pax7, a transcription factor with important functions in muscle regeneration, is protected from proteasome-dependent degradation when phosphorylated by CK2 (43).

Here we add HSJ1 to the list of the CK2 targets that are related to the proteasome function. We identified the HSJ1 sites where phosphorylation occurs by a hierarchical mechanism, and demonstrated that the presence of phosphate within the second UIM of HSJ1 reduces its binding to Ub-clients and its chaperone activity, without affecting the protein subcellular distribution. The identification of CK2 as the main HSJ1 phosphorylating agent was confirmed not only by the perfect match of the sites to the CK2 consensus, but also by the observation that different structurally unrelated CK2 inhibitors prevented phosphorylation.

Interestingly, the HSJ1 CK2 sites in UIM2 are conserved amongst different species (Supplementary Material, Fig. S3), although in mouse Ser247 is replaced by a His, and in two other cases Ser250 is replaced by acidic residues. At present, we do not know the exact contribution of each of these sites to the overall phosphorylation of human HSJ1 *in vivo*, although the *in vitro* analysis suggests a balance of 50% between S247 and S250. The presence of phosphorylatable residues at both Ser247 and Ser250 positions could be interpreted as an adaptive device, representing a mechanism to fine tune the function of UIM2. Our results obtained with separate mutations (Figs 6B and 7B) disclose only minimal differences on HSJ1 function. Ser250-to-Ala mutation also prevents Ser247 hierarchical phosphorylation, while in Ser247Ala mutant Ser250 is still phosphorylated; hence, the similar effects observed with the two mutants suggest a prominent functional role to Ser247. However, future analysis will be necessary to discriminate the full impact of each of these sites.

The effects of UIM phosphorylation in HSJ1 is the opposite of what reported for the SIM, where the CK2-dependent phosphorylation enhances the affinity for SUMO (33,34), and for UBA, the Ub-binding domain of p62 that increases its affinity for clients when phosphorylated by CK2 (beside displaying altered specificity) (35). However, the binding of SUMO to SIM (44), and of Ub to UBA (45) are due to a combination of hydrophobic and electrostatic interactions, while Ub typically interacts to UIMs mainly through hydrophobic bonds (46,47). It should be also considered that p62 is phosphorylated in many other sites that can modulate the final outcome.

The complex correlation between Ub-binding and phosphorylation is further highlighted by the observation that phosphorylation of ataxin-3 produces variable effects, depending on the UIM domain considered (31). Further studies will be necessary to fully understand the functional effects of HSJ1 phosphorylation by CK2 and the exact mechanism of UIM regulation.

The addition of HSJ1 among the numerous CK2 substrates has a relevance to neurodegenerative diseases. The importance of CK2 to neuronal functions and disease is supported by several findings. Already in 2000, Blanquet and colleagues reviewed data supporting a potential role of CK2 in neuronal function and implicated it in degenerative disorders (48) and this was extended later (49). More recently, it was found that CK2 phosphorylates and promotes stabilization and aggregation of ataxin3 (ATXN3), the pathogenic protein of spinocerebellar ataxia type 3 (SCA3) (31). HSJ1, here identified as a novel CK2 substrate, is related to protein aggregation and neurodegeneration, since it exerts a neuroprotective function by processing aggregation-prone proteins and promoting their delivery to the proteasome (1), preventing the seeding of aggregation (11) or refolding of mutant proteins (14,16). Our results, showing that CK2 inhibits HSJ1 function, suggest that CK2 inhibition might help enhance HSJ1 function in the clearance of Ub-protein aggregates and deserve attention in a therapeutic perspective. CK2 inhibitors, already under investigation as anticancer drugs, might in future also be applied to neurodegenerative conditions to prevent or reduce protein aggregation. Overexpression of HSJ1a has been shown to be protective in several models of neurodegeneration both in cells and *in vivo* by UIM dependent mechanisms (11,15). Our data suggest that simultaneous HSJ1a overexpression and CK2 inhibition could synergise the chaperone activity and further prolong neuronal survival.

In future, it will be worth investigating whether a competition occurs between Ub-proteins and CK2 for the recognition sequence on HSJ1 UIM2. This could mean that, under conditions where Ub-proteins tends to accumulate and an intense activity of HSJ1 is required, Ub-proteins mask the CK2 sites and prevent their phosphorylation, thus ensuring HSJ1 high efficacy. On the other hand, whenever CK2 is abnormally high, as usually occurs in cancer, it could prevail and block the regular clearance of Ub-proteins by HSJ1. This also suggests that a CK2-mediated link might exist between cancer and neurodegenerative diseases, and that any imbalance between CK2 expression and the machinery represented by HSJ1/Ub-clients/proteasome could be detrimental.

The focus of this work was HSJ1; however, UIMs are well-conserved domains, present in several proteins with diverse functions; we have preliminary results showing that indeed CK2 efficiently phosphorylates other UIM-containing proteins. Future work will be dedicated to understand whether CK2-mediated phosphorylation might be a general mechanism for regulating their functions; it is also conceivable that protein-specific elements contribute to modulate the effects of UIM phosphorylation, thus adding further complexity to the intersection between CK2 and Ub signalling.

## Materials and methods

### Antibodies

Anti-CK2 α was raised in rabbit against the sequence of the human protein at C-terminus [376–391], as previously described (50), anti-CK2 β was from Abcam; anti-pSer129 Akt was either produced in rabbit as in (28) or purchased from Abcam, anti-total Akt and anti-total HSJ1 were from Santa Cruz Biotechnologies, anti-myc, anti-tubulin and anti-actin from Sigma; anti-mono/poly-ubiquitinylated conjugates was from Enzo Life Sciences. Anti-pHSJ1 were produced in rabbits, as described below, and characterized as in Fig. 2 and Supplementary Material, Fig. S2.

### Protein purification

Human His-tagged HSJ1 WT and mutants cloned in pET-14b vectors were used to transform *E. coli* BL21(DE3) strain. The bacterial culture was grown to reach an Optic Density (OD) of 0.3–0.4 when protein synthesis was induced for 4 h by 100 mM Isopropil-b-D-1-tiogalattopiranoside (Sigma-Aldrich). Bacteria were then harvested by centrifugation and sonicated in bacterial lysis buffer (50 mM NaH_2_PO_4_ pH 8, 100mM NaCl, 0.01% Tween-20 and 2 mM DTT). Cell debris were discarded by centrifugation (35000 x g, 10 min) and HSJ1 was purified from the supernatant by His-selective affinity resin (Sigma-Aldrich) and eluted with 300 mM imidazole pH 8. Aliquots of the purified proteins were stored at −80°C in 10% glycerol, 2 mM DTT.

### *In vitro* phosphorylation assays of HSJ1 proteins and peptides

Recombinant purified human HSJ1 or peptides were incubated for 10min at 30°C with recombinant CK2 and [γ-^33^P] ATP in an appropriated phosphorylation buffer (50 mM Tris–HCl pH 7.5, 10 mM MgCl_2_, 50 µM [γ-P^33^] ATP at 2000 cpm/pmol (PerkinElmer) and 0.1 M NaCl). Phospho-reactions were stopped spotting peptides onto phospho-cellulose papers (Perkin Elmer) or by adding Laemmli buffer. Proteins were resolved onto SDS-PAGE, stained with Coomassie blue and analysed by digital autoradiography (CyclonePlus Storage Phosphor System, PerkinElmer).

### HSJ1 phospho-sites identification

#### Phospho-HSJ1 digestion

Recombinant HSJ1 was incubated with recombinant CK2 for 60 min at 30°C in a phosphorylation buffer suitable for the subsequent protein digestion (Phosphate Buffer pH 7.8, 10 mM MgCl_2_, 50 µM ATP and 0.1M NaCl). Disulfide bonds were then reduced by 5mM DTT for 30 min at 60°C followed by alkylation with 15mM iodoacetamide for 15 min at room temperature. Finally, the final volume was brought to 50 µL and HSJ1 was digested overnight at 37°C by Glu-C protease (Promega) as from manufacturer’s instruction. The reaction was then sent to mass spectrometry analysis.

#### Phosphopeptides enrichment and LC-MS/MS analysis

Enrichment of phosphorylated peptides was carried out as reported in (51). Briefly, samples were subjected to a phosphopeptide enrichment step using home-made TiO_2_ micro-columns and analysed by LC-MS/MS using a LTQ-Orbitrap XL mass spectrometer (ThermoFisher Scientific) coupled on-line with a nano-HPLC Ultimate 3000 (Dionex – ThermoFisher Scientific). Samples were loaded onto a 10 cm pico-frit column (75 μm I.D., 15 μm tip; New Objective) packed with C_18_ material (Aeris Peptide 3.6 μm XB-C18, Phenomenex) and separated using a 45 min linear gradient of ACN/0.1% formic acid (from 0% to 40% ACN in 25 min), at a flow rate of 250 nL/min. To increase phosphopeptides identification confidence, every sample was analysed three times with three different fragmentation methods (MS^2^, MS^3^, and MultiStage Activation), as detailed in (51).

Raw data files were analysed with a MudPit protocol against the Human section of the Uniprot database (version 20150107, 89706 sequences) with the software Proteome Discoverer 1.4 (ThermoFisher Scientific) interfaced to a Mascot search engine (version 2.2.4, Matrix Science). Enzyme specificity was set to V8-DE with up to 3 missed cleavages. Mass tolerance window was 10 ppm for parent mass and 0.6 Da for fragment ions. Carbamidomethylation of cysteine was set as fixed modification. Oxidation of methionine residues and phosphorylation of serine, threonine, and tyrosine were set as variable modifications. The algorithm PhosphoRS (52) was used to help in the assignment of the correct phosphorylation sites. False Discovery Rate (FDR) was calculated by Proteome Discoverer based on the search against the corresponding randomized database. Phosphopeptides identified with high (99%) confidence, were manually inspected for sequence and phosphorylation site confirmation.

### Site-directed mutagenesis

HSJ1 mutations were introduced by site-directed mutagenesis with the QuikChange II Site-Directed Mutagenesis Kit (Agilent Technologies) in accordance with the manufacturer’s protocol. Mammalian and bacterial vectors for human HSJ1 were as in (1,7). Custom-made primers were made by Sigma-Aldrich.

**Table:** 

Primer	SEQUENCE (5’-3’)
**Forward S247A**	**GCCTCATGCCCCTTGGACGCCGACCTCTCTGAGGATGAGG**
**Reverse S247A**	**CCTCATCCTCAGAGAGGTCGGCGTCCAAGGGGCATGAGGC**
**Forward S250A**	**CCCCTTGGACAGCGACCTCGCCGAGGATGAGGACCTGCAG**
**Reverse S250A**	**CTGCAGGTCCTCATCCTCGGCGAGGTCGCTGTCCAAGGGG**
**Forward S262A**	**GCAGCTGGCCATGGCCTACGCCCTGTCAGAGATGGAGGC**
**Reverse S262A**	**GCCTCCATCTCTGACAGGGCGTAGGCCATGGCCAGCTGC**
**Forward S250D**	**CCCCTTGGACAGCGACCTCGATGAGGATGAGGACCTGCAG**
**Reverse S250D**	**CTGCAGGTCCTCATCCTCATCGAGGTCGCTGTCCAAGGGG**

### Cell culture and treatments

All cells were cultured in an atmosphere containing 5% CO_2_; HEK293T (human embryonic kidney) cells and CHO (Chinese hamster ovary) cells were maintained in DMEM (Sigma), human BJ fibroblasts (ATCC, CRL-2522) in DMEM plus non-essential amino acids (Sigma) and SK-N-SH (human neuroblastoma) cells in DMEM/F-12 1:1 nutrient mixture (Sigma); media were supplemented with 10% (v/v) fetal bovine serum (FBS, Sigma), 2 mM L-glutamine (Sigma), 100 U/mL penicillin, and 100 mg/mL streptomycin (Sigma). Cell treatments with inhibitors were performed in the culture medium. Control cells were treated with equal amounts of the inhibitor solvent, which never exceeded 0.5% (v/v).

CX-4945 (Cylene Pharmaceuticals), CX-5011 (Glixx Laboratories), and TBB (kindly donated by Dr. Z. Kazimierczuk, Warsaw, Poland) were all dissolved in 10 mM stock solutions in DMSO (Sigma).

### Cell transfection, lysis and immunoprecipitation

Transient expression of HSJ1 WT and mutants was induced by transfecting CMV-Myc-Tag3a vectors into mammalian cell lines. Cells were plated into 6-well plates (2.5 × 10^5^ cells/well) the day before transfection and grown to about 70% confluence. HEK-293T cells were transiently transfected with 1 μg of plasmids by a standard calcium-phosphate procedure. The transfection mixture was removed after 16 h and cells were collected 48 h after transfection. Similarly, SK-N-SH cells were transiently transfected either on plates or 8-wells Permanox microscope slides (Sigma-Aldritch) with 1 μg of plasmids using Lipofectamine LTX Reagent (Life technologies) accordingly to the manufacturer's protocol. 48 h after transfection cells were collected or slides fixed for ICC analysis.

Cells were lysed with an ice-cold buffer containing 20 mM Tris–HCl, pH 7.5, 150 mM NaCl, 2 mM EDTA, 2 mM EGTA, 0.5% (v/v) Triton X-100, 2 mM dithiothreitol (DTT), protease inhibitor cocktail complete (Roche), 10 mM NaF, 1 μM okadaic acid (Enzo Life Sciences), and 1 mM Na vanadate. After 60 min incubation on ice, the lysates were centrifuged at 16000 x g for 30 min, at 4°C and the supernatants were subjected to the Bradford quantification assay before SDS-PAGE separation and western blot analysis.

#### ^32^P[phosphate] cell labelling

HEK-293T cells were transfected with HSJ1 WT and mutants by a standard calcium phosphate protocol as described above. 44 h after transfection cells were washed twice with PBS (Sigma) and incubated with phosphate-free DMEM (Sigma) supplemented with 50 µCi/mL of ^32^P[phosphate] for 3 h. If required, cells were treated with 10 µM CX-4945 for the same length of time. Cells were then harvested and lysed before being subjected to (co)immunoprecipitation protocol, digital autoradiography and western blot analysis.

### Western blotting

Equal amounts of proteins from cell lysates were analysed onto 11% SDS-PAGE and blotting on PVDF membranes (Immobilon-P Millipore) using the Lightning Blotter Transfer System (Perkin Elmer) and buffers as from manufacturer’s instructions. Dried membranes were then washed with 1% (w/v) bovine serum albumin (Sigma) in TTBS buffer (Tris–HCl 50 mM pH 7.5, NaCl 50 mM, 0.1% Tween-20) and incubated with the indicated antibodies. Membranes were then incubated with secondary HRP-conjugated antibodies (Perkin Elmer) for 1 h and bands were detected by a chemiluminescence solution composed by 2.25 ml H_2_O, 250 μl 1 M Tris, pH 9.35, 1 μl H_2_O_2_ and 2.5 ml of a luminol solution (prepared with luminol 78 mg and p-iodophenol 95 mg dissolved in 100 ml 0.1 M Tris pH 9.35) plus 30% (w/v) BSA. Finally, bands were visualized with the Kodak Image Station 4400MM PRO and quantified with the Kodak 1D Image software.

### Stable transfection

CHO inducible cell lines were produced using the Flp-in T-REx system by following the manufacturer’s instructions (Thermo-Scientific). eGFP-tagged HSJ1a or HSJ1b in the pcDNA5/FRT/TO was received as a gift from Prof. HH Kampinga (Groningen; (53)) and used to produce inducible stable cells lines expressing HSJ1 proteins under the control of tetracyline.

### Peptide synthesis

The synthetic peptides PLDSDLSEDED-betaAla-RRR, PLDSDL�pSED�ED-betaAla-RRR PLDSDLSEDED-betaAla-C, PLDSDLpS�ED�ED-betaAla-C, PLDpSDLpSEDED-betaAla-C were synthesized by solid-phase technique using a multiple peptides synthesizer (SyroII, MultiSynTech GmbH) on a pre-loaded Wang resin (100–200 mesh) with Fmoc-*N*ε-*tert*-butyloxycarbonyl-l-lysine (Nova�biochem). The fluoren-9-ylmet�hoxycarbonyl (Fmoc) strategy was used throughout the peptide chain assembly, utilizing O-(7-azabenzotriazol-1-yl)-N’,N’,N′,N′-tetramethyluronium hexafluorophosphate (HATU) as coupling reagent. The side-chain protected amino acid building blocks used were: N-α-Fmoc-Nω-(2,2,4,6,7-pentamethyldihydrobenzofuran-5-sulfonyl)-L-argi�nine, *N*-α-Fmoc-β-*tert*-butyl-l-aspartic acid, N-α-Fmoc-γ-tert-butyl-L-glutamic acid, N-α-Fmoc-O-tert-butyl-L-serine, N-α-Fmoc-S-trityl-cystine, and N-α-Fmoc-O-benzyl-phospho-L-serine. Cleavage of the peptides was performed by incubating the peptidyl resins with trifluoroacetic acid/H2O/triisopropylsilane (95%/2,5%/2,5%) for 2.5 h at 0°C. Crude peptides were purified by reverse phase HPLC on a preparative column (Prep Nova-Pak HR C18). Molecular masses of the peptide were confirmed by mass spectroscopy on a MALDI TOF-TOF using an Applied Biosystems 4800 mass spectrometer.

### Phospho-antibodies production and purification

Antibodies specific to phospho sites Ser-247 and Ser 250 were generated. New Zealand rabbits were immunized with the following synthetic peptides (from human HSJ1 sequence [244–254]) PLDSDLSEDED-betaAla-C, PLDSDLpSEDED-betaAla-C and PLDpSDLpSEDED-betaAla-C coupled with maleimide-activated keyhole limpet hemocyanine (KLH) or bovine serum albumin (BSA) (1:1 w/w) through the C-terminal cysteine. Rabbits were injected four times at 3-week intervals, with 0,5 mg of peptide-proteins conjugates emulsified with Freund’s adjuvant (1:1 v/v). Antisera were purified using an immobilized peptide affinity resin (Sulfo Link Coupling Gel, Pierce) according to manufacturer’s instructions.

### Ub-protein binding analysis

Cells were lysed in Co-IP Buffer (25 mM MOPS pH 7.2, 100 mM KCl, 5mM EDTA, 0.5% Tween-20), then 0.5–1 mg of total protein lysate was incubated with anti-myc antibody for 2 h followed by incubation with Protein G PLUS-Agarose resin (Santa Cruz Biotechnology) for 45 min. The resin was then washed twice with wash buffer (25 mM MOPS pH 7.2, 100 mM KCl, 5mM EDTA). Myc-tagged HSJ1 and co-precipitated proteins were eluted with loading buffer and subjected to gel electrophoresis followed by western blot analysis with anti-Ub antibody.

### Statistical analysis

Statistical analysis was performed with the Software Graphad Prism and significance determined using the unpaired, two-tailed, Student’s *t*-test. All data were collected from at least 3 independent experiments; representative experiments are shown. Graphs show quantifications, SEM and significances (*P ≤ 0.05, ** *P* ≤0.01, ***P ≤ 0.001).

### Immunofluorescence experiments

For immunofluoescence staining, coverslips were washed twice with PBS and fixed in 4% paraformaldehyde for 10 min. Cells were then permeabilized with 0.5% Triton X-100 for 10 min, at room temperature. Then, microscope slides were incubated in blocking buffer (3% bovine serum albumin (BSA) and 10% normal donkey serum in PBS) for 1 h before incubation with primary antibodies for 1 h at room temperature. Species-specific anti-IgG Alexa Fluor 488 secondary antibodies were used and nuclei were stained with DAPI (Sigma). Slides were mounted with fluorescence mounting medium (Dako) and images were acquired with the 63X objective of Zeiss LSM700. Scale bars are 50 µM.

### Luciferase assay

SK-N-SH cells were seeded in 96-well plates and transfected at 80% confluence with TransIT-LT1 Transfection Reagent (Mirus). Each well was transfected with 20 ng of HSJ1 WT and mutants, and 10 ng of the pBK-CMV, a cytomegalovirus (CMV)-promoter-driven firefly luciferase reporter vector.

Luciferase was expressed in stably HSJ1-transfected CHO cells as described above. HSJ1 synthesis was induced 3 h post transfection by 3 µg/ml tetracycline (Invitrogen). If required, after 21 h cells were treated with 10 µM CX-4945. 24 h after transfection, luciferase activity was measured on an Orion L Microplate Luminometer (Titertek Berthol) with a dual-luciferase reporter assay system (Dual-Glo Luciferase Assay System, Promega) in accordance with the manufacturer’s protocol.

## Supplementary Material

Supplementary Material is available at *HMG* online.

## Supplementary Material

Supplementary Data
