# Supplementary material for: Protein kinase CK2 modulates HSJ1 function through phosphorylation of the UIM2 domain
Source: Hum Mol Genet. 2016 Dec 28;26(3):611–23. doi: 10.1093/hmg/ddw420 (PMC5409130; doi:10.1093/hmg/ddw420)
Supplement: Supplementary Data [file ddw420_Supp.zip › ddw420-suppl_data/HMG-2016_D_01055_R1_OTTAVIANI_MAT_SUPPL.docx]

**SUPPLEMENTARY MATERIALS**

**RESULTS**

**Specificity of the phospho-HSJ1 antibodies**

We applied the SPR technology to assess the recognition of the phospho-HSJ1 antibodies toward the mono- and bis-phosphorylated peptides reproducing pSer250 and pSer250/pSer247 HSJ1 sequence, respectively. The peptides were immobilized on a chip surface, and the antibodies flowed in solution over the chip. The results (Fig. SM2A) clearly showed that the pSer250 peptide was strongly recognized, with a calculated K_D_ of 7.65x10^-9^ M + 5.77x10^-10^; on the contrary, the signal towards pSer250/pSer247 peptide was much weaker, and the KD was 8.29x10^-8^ + 4.92x10^-9^ M.

Since the peptide immobilization could affect the affinity (55), we also applied a competition protocol, where the antibodies were injected over the chip in the presence of increasing concentrations of the pSer250 peptide, or pSer250/pSer247 peptide, to assess their ability to prevent the binding to the pSer250 peptide. This analysis allowed the determination of IC_50_ value for each peptide (Fig. SM2B), which confirmed that the mono-phosphorylated peptide is the best recognized, while, also in solution, the bis-phosphorylated peptide has lower affinity.

**METHODS**

**Surface plasmon resonance analysis**

A BiacoreTM T100 (GE Healthcare) instrument was used. HSJ1 pSer250 (P-peptide, PLDSDLpSEDED-βA-Cys) or pSer247/pSer250 (PP-peptide, PLDpSDLpSEDED-βA-Cys) were immobilized by thiol coupling on a CM5 (series S) sensor chip (carboxymethylated dextran surface) to a final density of 171 (P-peptide) and 174 (PP-peptide) resonance units (RU). A flow cell with no immobilized peptide was used as control. Antibody binding analysis was carried out in a running buffer consisting of 10 mM HEPES, pH 7.4, 150 mM NaCl, 0.001% (v/v) Tween-20, applying a flow rate of 10 μl/min, with 420 s antibody injection time, followed by 420 s dissociation time. For kinetics experiments, a Biacore method program was used. It included a series of three start up injections (running buffer), zero control (running buffer) and 5 different concentrations. Peptide competition experiments were performed by injecting 2 nM antibodies over the sensor chip at a flow rate of 20 μl/min, 180 s injection time, 180 s dissociation time, in the presence of increasing concentrations (1 to 200 nM) of P-peptide or PP-peptide. In all cases, the chip surface was regenerated with 30 s injections of 1 M NaCl plus 30 s injection of 0.1 M HCl; this treatment restored the baseline to the initial RU value. Each sensorgram (time-course of the surface plasmon resonance signal) was corrected for the response obtained in the control flow cell and normalized to baseline. The kinetic data were analysed using the 2.0.3 BIAevaluation software (GE Healthcare). Curves were fitted with the classical Langmuir 1:1 model; the quality of the fits was assessed by visual inspection of the fitted data and their residual, and by chi-square values. Two independent experiments for each analysis were performed.

**LEGENDS**

**Table SM1**

The table lists all peptides identified with high confidence by LC-MS/MS after the phosphopeptide enrichment procedure. All details regarding peptide identification (sequence, Mascot score, expectation value, number of missed cleavages, m/z, delta mass) are reported.

**Fig. SM1 Mass spectrometry identification of CK2 sites on HSJ1**

A. Annotated MS/MS spectra relative to the phosphopeptides DLQLAmAYSLpSE (Panel A) and QQPSVTSRSGGTQVQQTPAScPLDpSDLpSE (Panel B) and tables with all identified fragment ions.

**Fig. SM2. Kinetics of the phospho-HSJ1 antibody interactions by means of SPR signal.**

A. Increasing concentration of the antibody (μM values indicated) were injected over a sensor chip where pSer250 peptide (left panel) or pSer247/pSer250 peptide (right panel) were immobilized in a Biacore T100 instrument (see the Methods for details). Surface plasmon resonance (SPR) signal is shown as sensorgram (time course of the response) reported in resonance units (RU). Each sensorgram has been subtracted of the corresponding signal produced on a control surface and normalized to baseline. 0 concentrations corresponded to dilution buffer. B. 0.002 μM phospho-HSJ1 antibody was injected over the pSer250 peptide immobilized on the chip, in the presence of increasing concentrations of pSer250 peptide or pSer247/pSer250 peptide. The RU signals at the end of the binding time (relative response subtracted of the control signal) for each condition were plotted and the calculation of the IC50 was performed by GraphPad software.

**Fig. SM3. Alignment of UIM2 sequence of *DNAJB2* from representative species.**

Species are indicated in the left column; the right column shows SwissProt/TrEMBL access numbers. Dark grey, invariant residues; light grey, conservative substitutions; arrows indicate positions corresponding to CK2 sites of human sequence identified in this work.
