# Supplementary figures and images for: Protein kinase CK2 modulates HSJ1 function through phosphorylation of the UIM2 domain
Source: Hum Mol Genet. 2016 Dec 28;26(3):611–23. doi: 10.1093/hmg/ddw420 (PMC5409130; doi:10.1093/hmg/ddw420)

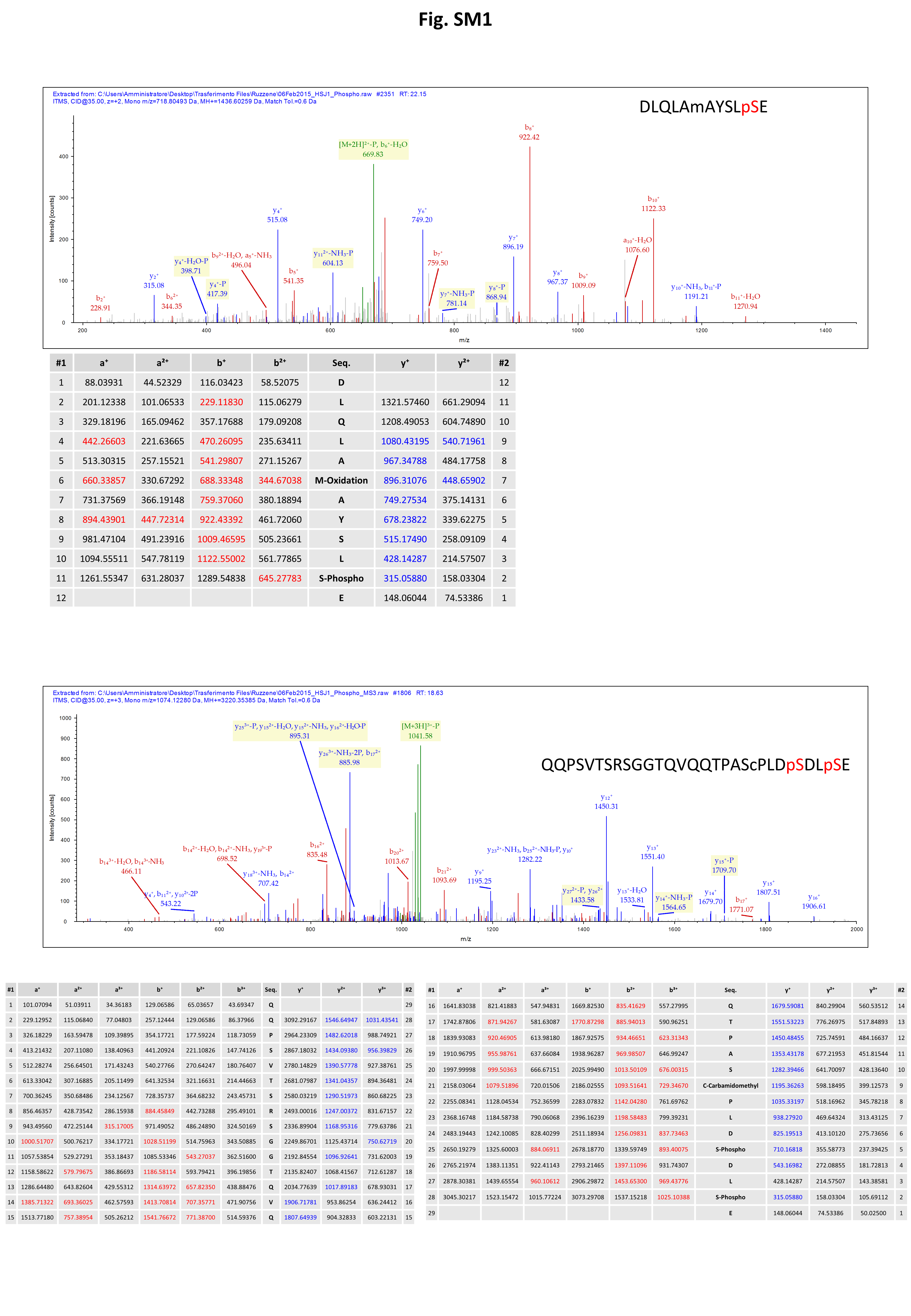

Supplement: Supplementary Data [file ddw420_Supp.zip › ddw420-suppl_data/Fig SM1 OTTAVIANI.tif]

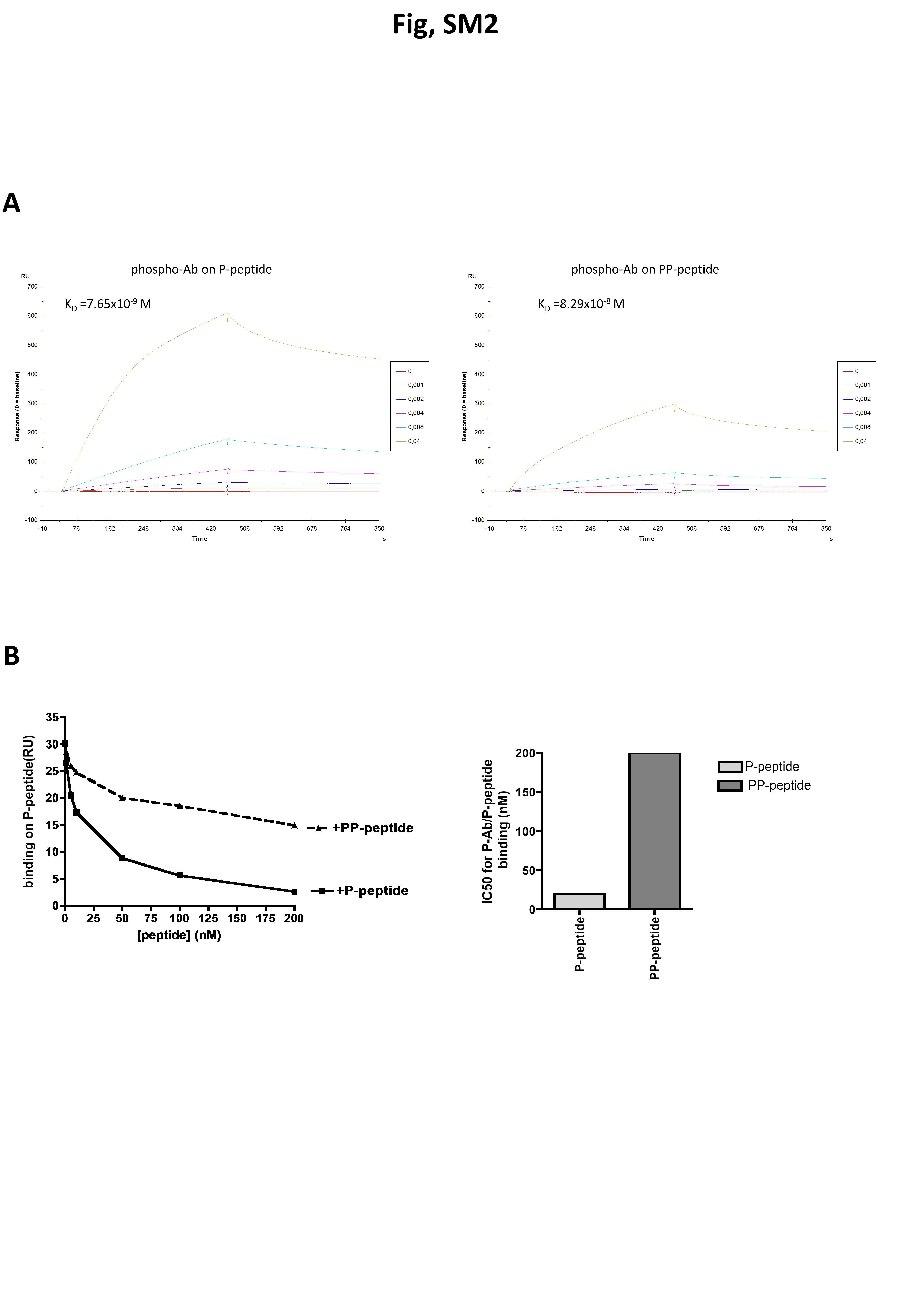

Supplement: Supplementary Data [file ddw420_Supp.zip › ddw420-suppl_data/Fig SM2 OTTAVIANI.tif]

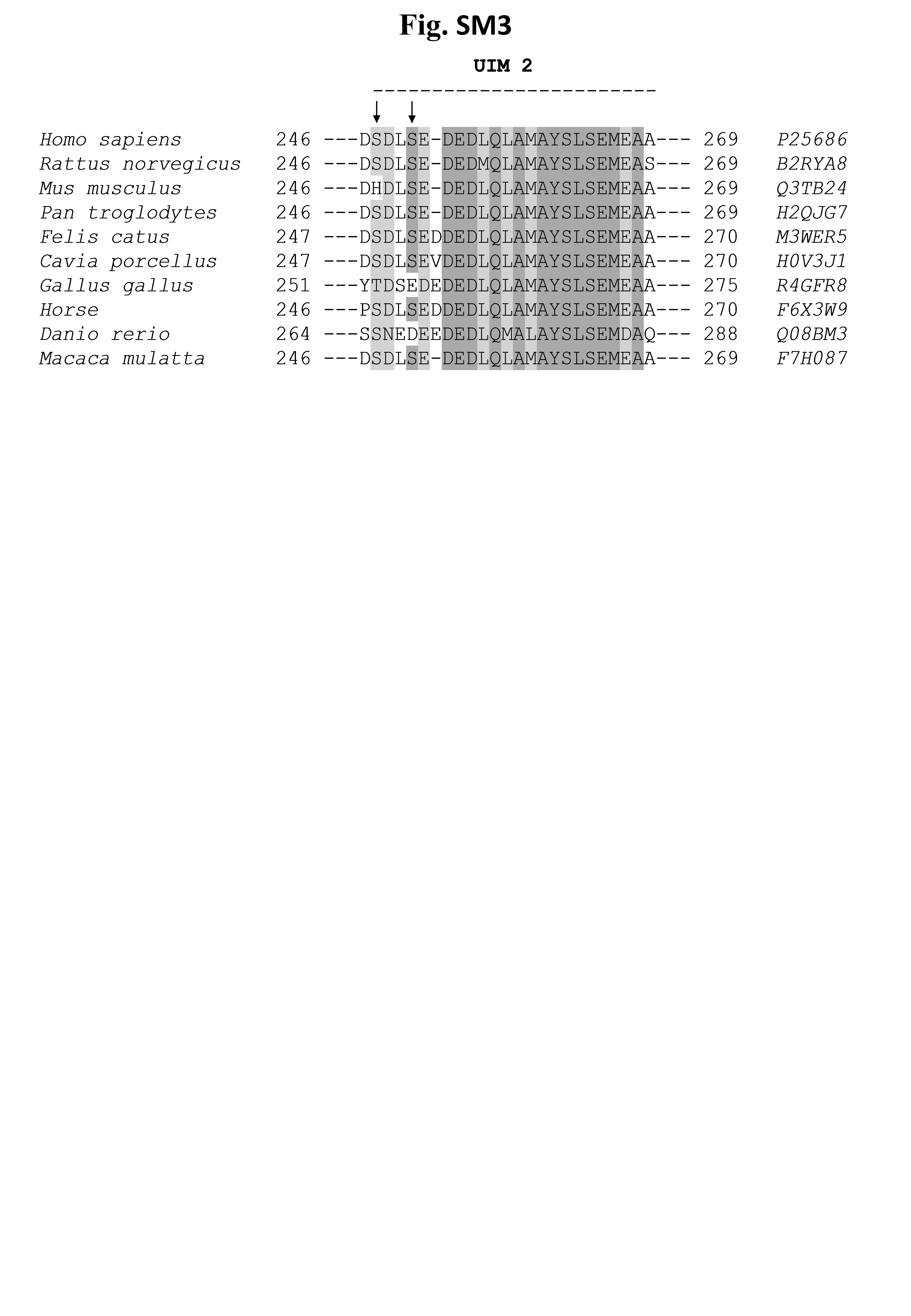

Supplement: Supplementary Data [file ddw420_Supp.zip › ddw420-suppl_data/Fig SM3 OTTAVIANI.tif]
